# Invisible surfaces enabled by the coalescence of anti-reflection and wavefront controllability in ultrathin metasurfaces

**DOI:** 10.1038/s41467-021-24763-9

**Published:** 2021-07-26

**Authors:** Hongchen Chu, Haoyang Zhang, Yang Zhang, Ruwen Peng, Mu Wang, Yang Hao, Yun Lai

**Affiliations:** 1 National Laboratory of Solid State Microstructures, School of Physics, and Collaborative Innovation Center of Advanced Microstructures, Nanjing University, Nanjing, China; 2 School of Electronic Engineering and Computer Science, Queen Mary University of London, London, UK

**Keywords:** Metamaterials, Sub-wavelength optics

## Abstract

Reflection inherently occurs on the interfaces between different media. In order to perfectly manipulate waves on the interfaces, integration of antireflection function in metasurfaces is highly desired. In this work, we demonstrate an approach to realize exceptional metasurfaces that combine the two vital functionalities of antireflection and arbitrary phase manipulation in the deep subwavelength scale. Such ultrathin devices confer reflection-less transmission through impedance-mismatched interfaces with arbitrary wavefront shapes. Theoretically and experimentally, we demonstrate a three-layer antireflection metasurface that achieves an intriguing phenomenon: the simultaneous elimination of the reflection and refraction effects on a dielectric surface. Incident waves transmit straightly through the dielectric surface as if the surface turns invisible. We further demonstrate a wide variety of applications such as invisible curved surfaces, “cloaking” of dielectric objects, reflection-less negative refraction and flat axicons on dielectric-air interfaces, etc. The coalescence of antireflection and wavefront controllability in the deep subwavelength scale brings new opportunities for advanced interface optics with high efficiency and great flexibility.

## Abstract (web summary)

Though existing metamaterial antireflection coatings have been optimized in terms of thickness and functionality, these coatings do not provide phase control in the deep subwavelength scale. Here, the authors report multi-layered metasurfaces that provide both antireflection and phase control.

## Introduction

Metasurfaces^1–3^ are ultrathin wave-functional devices composed of arrays of subwavelength resonators that can interact with electromagnetic waves ranging from microwaves to optics. By introducing spatially varying optical response into these miniaturized resonators, we can arbitrarily control both wavefront and polarization of the refracted and reflected beams with accuracy and precision^4–10^. Metasurfaces have therefore been applied to numerous novel applications including ultrathin lensing^11–17^ and holography^18–21^, invisibility and illusionary effects^10,22–27^, propagating-to-evanescent wave conversion^28–30^, photonic spin Hall effect^31–33^, and generation of structured light field^34,35^, etc. So far, the pioneering metasurface demonstrating the generalized Snell’s law^4,5^ suffers from low efficiency. Reflection-type metasurfaces^10,29,30^ can achieve high reflectivity by incorporating a reflecting mirror with the metasurface. However, transmission-type metasurfaces enabling high transmission with controllable phase is much more challenging. Various approaches including the theory of Huygens’ metasurface^36–38^, and structures with high symmetry^8,27,28^ have been applied to enhance the transmittance. Nevertheless, almost all previous high-efficiency transmission-type metasurfaces were designed for a single background medium such as free space, or free space with a low-index substrate like silica. While on the interfaces between materials with more different indexes or impedances, substantial reflection would occur, which can significantly reduce the efficiency of metasurfaces and may even disable the designed functionalities. This inherent reflection severely hinders the application of high-efficiency metasurfaces on impedance-mismatched interfaces.

In order to eliminate the reflection on dielectric surfaces, the traditional approach is to apply antireflection coatings that are based on the principle of destructive interference^39,40^. Classical quarter-wavelength antireflection coatings require a particular refraction index $n=\sqrt{{n}_{0}{n}_{g}}$ and a considerable thickness *d* = *λ*/4, where *n*_0_, *n*_*g*_, and *λ* are the refraction indexes of the two media and the wavelength in the antireflection layer. Multi-layered antireflection coatings require even much larger thicknesses. Metamaterials, which exhibit unusual effective medium parameters absent in nature, have been applied in the design of advanced antireflection coatings^41–43^. Pioneering works have shown that the thickness of the metamaterial antireflection coatings can be significantly reduced to a scale far below the limit of traditional ones^41^. The functionality of antireflection can also be extended to operate within a wide range of incident angles and frequencies^43^. However, none of the previous anti-reflection coatings based on metamaterials has considered the controllability of the transmission phase. As we shall prove later, if a single layer of metamaterial satisfies the effective medium approximation, the principle of destructive interference would limit the transmission phase to ±*π*/2, thereby disabling the phase control ability. This contradiction hinders the coalescence of antireflection and phase control abilities in the deep subwavelength scale.

In this work, we demonstrate that multi-layered metasurfaces can solve the above fundamental contradiction between antireflection and phase control, and thus merge the two vital functionalities in a deep subwavelength scale. Such anti-reflection metasurfaces allow near-perfect transmission through originally impedance-mismatched interfaces between different materials, with arbitrarily chosen wavefront shapes. The physical origin in the coalescence of the originally conflicting functionalities lies in the multiple interference effect and the abundance of the degrees of freedom in the multi-layered metasurfaces, which are far beyond their one-layer or two-layer counterparts. A wide variety of high-efficiency interface devices can be designed based on the coalesced functionalities. As a demonstration, we have designed and experimentally verified a unique metasurface composed of three-layer metallic patterns that allows obliquely incident beams to transmit straightly through a dielectric surface, with neither reflection nor refraction. In other words, the dielectric surface is made invisible to the incoming waves. Furthermore, we show that such anti-reflection metasurfaces can realize invisible corrugated surfaces, “cloaking” of dielectric objects, reflection-less negative refraction, and flat axicons in dielectrics, etc. Our work extends the concept of invisible surface^10,22–26^, which was limited in reflection, to the transmission geometry. These versatile devices demonstrate a great potential for such metasurfaces to improve the efficiency and increase the ways of coupling between materials with substantial contrast.

## Results

### Contradiction between antireflection and phase manipulation in traditional approaches

**Fig. 1:**
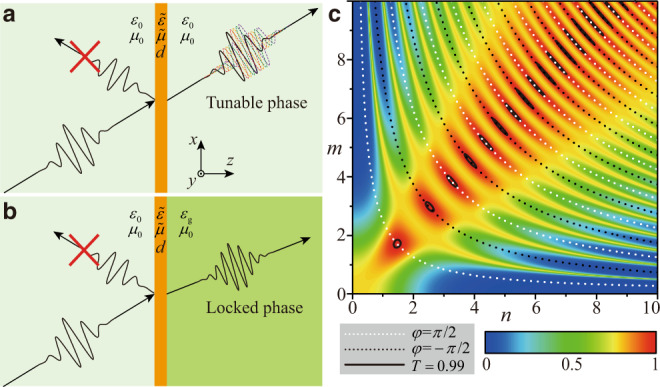
Locked transmission phase in a homogeneous antireflection coating. **a** Schematic diagram of a layer of anisotropic homogeneous medium with thickness *d*, relative permittivity $\tilde{\varepsilon }$ and permeability $\tilde{\mu }$ in free space. An oblique incidence with the incident angle *α* is considered. **b** Schematic diagram of a layer of anisotropic homogeneous medium located on the interface between the free space and a dielectric with relative permittivity ${\varepsilon }_{g}$. **c** Calculated transmittance *T* as a function of $m=\sqrt{{\varepsilon }_{y}-\,\sin^{2} \alpha/{\mu}_{z}}$ and $n=\sqrt{{\mu}_{x}}$, where ${\mu}_{x}$, ${\mu }_{{{{\rm{z}}}}}$ and ${\varepsilon }_{y}$ are the *x* and *z* components of the relative permeabilit*y* and the *y* component of relative permittivity of the homogenous layer. White and black dashed lines depict the contour plots of transmission phases of $\pi /2$ and $-\pi /2$, respectively. Black solid lines represent contour plot of the transmittance of 0.99. Here, *d*, $\alpha$and ${\varepsilon }_{g}$ are set as $0.1{\lambda }_{0}$, 45° and 4.4, where ${\lambda }_{0}$ is the wavelength in free space.

Firstly, we demonstrate that the conflict between antireflection and phase manipulation generally exists for one layer of traditional anti-reflection coating described by the effective medium approximation. The medium is assumed to be passive and lossless and the interface is in the *x-y* plane. Without loss of generality, we consider transverse-electric (TE) polarization with electric field along the *y* axis, as shown in Fig. 1a. The reflection and transmission coefficient of the slab can be derived through the Transfer Matrix Method (TMM), which gives

1a $$r=\frac{{q}_{0}\,\cos \,\varphi -{q}_{0}{q}_{g}(in/m)\sin \,\varphi +(im/n)\sin \,\varphi -{q}_{g}\,\cos \,\varphi }{{q}_{0}\,\cos \,\varphi -{q}_{0}{q}_{g}(in/m)\sin \,\varphi -(im/n)\sin \,\varphi +{q}_{g}\,\cos \,\varphi },$$

1b $$t=\frac{2{q}_{0}}{{q}_{0}\,\cos \,\varphi -{q}_{0}{q}_{g}(in/m)\sin \,\varphi -(im/n)\sin \,\varphi +{q}_{g}\,\cos \,\varphi }.$$

In the above formulas, ${q}_{0}=\scriptstyle\sqrt{{\varepsilon }_{0}{\mu }_{0}-{\sin }^{2}\alpha }/{\mu }_{0}$,${q}_{g}=\scriptstyle\sqrt{{\varepsilon }_{g}{\mu }_{g}-{\sin }^{2}\alpha }/{\mu }_{g}$ and $\varphi =mnd{k}_{0}$, where $m=\scriptstyle\sqrt{{\varepsilon }_{y}-\,\sin^{2} \,{\alpha }/{\mu }_{z}}$, $n=\sqrt{{\mu }_{x}}$, and ${k}_{0}$, *d* and $\alpha$ are the wave number in free space, the thickness of the slab and the incident angle, respectively. $\varepsilon$ and *μ* depict the relative permittivity and permeability. The subscripts *0* and *g* represent the background materials in the sides of incidence and transmission, respectively. The subscripts *x*, *y*, and *z* represent the components in the corresponding directions. In a homogeneous background (*i.e*. ${q}_{g}={q}_{0}$), as shown in Fig. 1a, there are two sets of solutions for zero reflection $R={|r|}^{2}=0$, and total transmission $T={|t|}^{2}=1$. One solution is $\sin \,\varphi =0$, which corresponds to the Fabry–Pérot resonances. By submitting $\sin \,\varphi =0$ into Eq. (1b), it can be immediately obtained that the transmission phase is locked at 0 or *π*. The other solution is obtained as ${(m/n)}^{2}={q}_{0}^{2}={q}_{g}^{2}$, which indicates that the impedance of the slab is equal to that of the background. In this case, the transmission phase is equal to the phase accumulation in the slab and therefore can cover a full range of 2*π*. However, when the background media are asymmetric (i.e. ${q}_{0}\;\ne\; {q}_{g}$), as shown in Fig. 1b, the only solution for $R=0$ and $T=1$ is $\cos \,\varphi =0$ and ${(m/n)}^{2}={{q}}_{{0}}{{q}}_{{g}}$. In this case, the transmission phase is locked at $\pi /2$ or $-\pi /2$. For $\alpha =0$, this condition reduces to the well-known quarter-wavelength condition of antireflection coatings^44^. The other solution with flexible transmission phase disappears in this model with the asymmetric backgrounds. In Fig. 1c, we plot the calculated *T* versus the parameters *m* and *n*, where *d*, *α*, and ${\varepsilon }_{g}$ are set as $0.1{\lambda }_{0}$, 45°, and 4.4, respectively. ${\lambda }_{0}$ is the wavelength in free space. The black solid lines represent the contour plot of *T* = 0.99. The white and black dashed lines depict the contour plots of transmission phases of *π/*2 and −*π/*2, respectively. It can be seen that high transmittance can only be obtained when the transmission phase is either *π/*2 or −*π/*2, which is consistent with the above theoretical analysis.

In order to solve the above contradiction between antireflection and phase control abilities, we must break the symmetry of the system and introduce more degrees of freedom. In such cases, the metasurface can no longer be approximated by an effective medium. We note that previously, ABA structures with spatial inversion symmetry were found to support high transmission and controllable transmission phase in a symmetric background^28^. Inspired by this type of three-layer structures, we explore the possibility of breaking the spatial inversion symmetry and using asymmetric three-layer ABC structures to achieve antireflection and phase manipulation simultaneously. Compared with ABA structures, ABC structure clearly exhibits more degrees of freedom and advantages for asymmetric backgrounds.

### Design of metasurface components with antireflection and phase manipulation

**Fig. 2:**
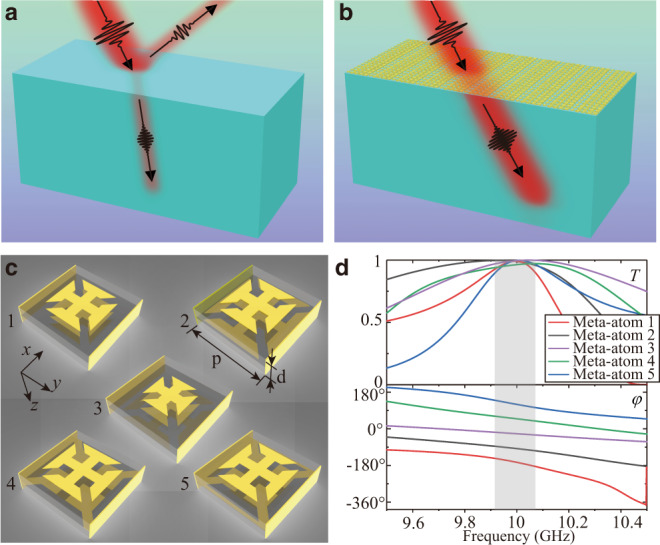
Meta-atoms of the gradient antireflection metasurface (GAM) for “invisible surface”. **a** Schematic diagram of the reflection and refraction occurring on a dielectric surface under oblique incident waves. **b** Schematic diagram of the elimination of both reflection and refraction on the dielectric surface by utilizing a GAM. **c** Structures of the five designed meta-atoms, which are all composed of 3-layer metallic patterns separated by two 1-mm-thick dielectric spacers. The side length and thickness of meta-atoms are, respectively, $p=7.73mm$ and $d=2mm$. Metallic sheets are used on the left and right sides of each meta-atom to suppress the mutual coupling between neighboring meta-atoms. **d** Simulated spectra of transmittance (upper panel) and transmission phase (lower panel) for the five designed meta-atoms on the interface between the free space and a dielectric material with its relative permittivity of 4.4. The gray shadow depicts a frequency range from 9.92 to 10.07 GHz, where the transmittances of all five meta-atoms are >90%.

In order to demonstrate the intriguing consequences of the coalescence of antireflection and phase control abilities, we demonstrate an unique application of making dielectric surfaces invisible in the transmission geometry. Previously, the realization of invisible corrugated surfaces by using reflection-type metasurfaces from microwaves to optical frequencies^10,22–26^ has attracted great interests in the field of electromagnetic wave. However, all those works were limited in the reflection geometry, where the efficiency is not an issue. In the transmission geometry, invisible surfaces are much more challenging to achieve because it requires the simultaneous elimination of the reflection and refraction effects. Antireflection metasurfaces with wavefront controllability are thus necessary. The concept of this idea is shown in Fig. 2. Figure 2a shows the normal reflection and refraction occurring on a flat dielectric surface. Figure 2b shows the function of the desired metasurface, which can suppress the reflection and bend the angle of refraction to the incident angle simultaneously. The surface is thus made invisible. Here, we note that the invisibility effect works only for a narrow range of incident angles and frequencies, which is currently a general limitation for metasurfaces cloaks. In order to achieve this invisible surface, the metasurfaces should be composed of antireflection meta-atoms with gradient transmission phases, which are thus denoted as gradient antireflection metasurfaces (GAMs).

The five meta-atoms of the GAM that bestows the invisible surface are shown in Fig. 2c. They are composed of three layers of metallic patterns separated by thin dielectric spacer. The length and thickness of the meta-atoms are $p=7.73mm$ and $d=2mm$, respectively. More detailed geometric parameters are shown in Supplementary Note 1 and Supplementary Fig. 1. By using the Finite-Difference Time-Domain (FDTD) simulations, we have calculated the transmittance and transmission phase spectra of such meta-atoms on the interface between the free space and a dielectric material with its relative permittivity of 4.4, as shown in Fig. 2d. It is observed that at the working frequency of *f*_0_ = 10 GHz, these meta-atoms exhibit both high transmittance and gradually changing transmission phase. The transmittance of these five meta-atoms at *f*_0_ are, respectively, 99.0%, 99.0%, 99.5%, 96.3%, and 99.8%, and the corresponding transmission phases are, respectively, −162.0°, −89.3°, −18.9°, 56.5°, and 124.6°. We note that in this design, metallic sheets have been applied to suppress the coupling between each meta-atom with its neighbors. The impact of the metallic sheets is described in details in Supplementary Note 2 and Supplementary Figs. 2–3. We also emphasize that despite of the three-layer design, the total thickness of the meta-atoms is only ${\lambda }_{0}/15$ (*λ*_0_ is the working wavelength), i.e., in the deep subwavelength scale.

### Numerical and experimental demonstrations of the invisible surfaces

**Fig. 3:**
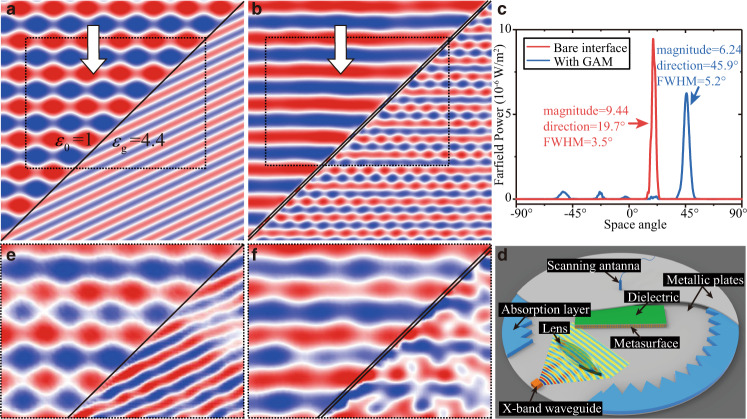
Simultaneous elimination of the reflection and refraction effects on a dielectric surface by the GAM. **a**, **b** Simulated ${E}_{z}$ field distributions for a dielectric surface without (**a**) and with (**b**) the designed GAM under illumination of a TE-polarized wave with the incident angle of $45^\circ$. **c** Simulated far-field radiation power pattern in the half-infinite space of dielectric material. **d** Schematic diagram of the experimental setup. **e**, **f** Experimentally measured ${E}_{z}$ field distributions for the dielectric surface without (**e**) and with (**f**) the GAM in the regions marked by the dotted box shown in (**a**) and (**b**), respectively.

By arranging the five meta-atoms sequentially, we constructed a supercell of the metasurface. By periodically replicating this supercell on the dielectric surface, we obtain the GAM that can make the dielectric surface invisible. The supercell has a length of *L* = 5*p*, which imposes a phase gradience of $\xi =2\pi /L$ to the transmitted waves. The parallel component of the wave vector of the transmitted waves is changed into ${k}_{t}^{//}={k}_{i}^{//}+\xi =\,\sin (\beta ){n}_{g}{k}_{0}$, which determines the angle of refraction. By setting ${k}_{i}^{//}=\,\sin (\alpha ){k}_{0}$ and $\beta =\alpha$, the angle of refraction can be made equal to the incident angle, hence eliminating the refraction effect. In this design, we set ${\varepsilon }_{g}=4.4$ and $\alpha =45^\circ$. Full wave simulations are performed by FDTD simulations for both cases with and without the GAM. Before the GAM is applied, both the reflection and refraction on the bare dielectric surface are evident, as shown in Fig. 3a. The ripple of wavefront in free space indicates the interference between the incident and reflected waves. The observed refraction angle *β* = 19.7° is consistent with the Snell’s law.

However, when the GAM is applied, as shown in Fig. 3b, the ripple of wavefront disappears in the free space, indicating that the reflection is eliminated. Furthermore, in the dielectric region, the wavefront indicates that the direction of transmission is changed to $\beta =45^\circ =\alpha$, i.e., the same angle as the incidence. The small fluctuation in the wavefront of transmitted waves is mainly attributed to the discretization of the metasurface as well as the coupling between neighboring units, as has been previously observed in other metasurfaces^27,29^. In the far field, the power radiation patterns are calculated for the cases with and without the GAM, as shown in Fig. 3c. The transmitted waves are evidently deflected to mainly propagate in the same angle as the incidence. By integrating the power flow, we find the transmittance in the direction of 45° occupies 76.3% of the total incident power, and the reflectance is dramatically reduced from 22.3% to 4.1%. The bandwidth of the GAM is roughly 0.15 GHz (~1.5% at 9.92 GHz to 10.07 GHz, as indicated by the gray shadow area in Fig. 2d), where the transmittances of all meta-atoms are greater than 90%. The simulated field distributions of this GAM at 9.92 GHz and 10.07 GHz are included in Supplementary Note 3 and Supplementary Fig. 4, which proves the bandwidth of the “invisible surface” phenomenon.

We further experimentally verify this intriguing phenomenon of invisible surface in the transmission geometry via microwave experiments. A rectangular dielectric slab of *ε*_*g*_ = 4.4 is placed inside a parallel metal plate waveguide. An X-band waveguide and a convex lens are used to generate a TE-polarized plane wave inside the waveguide, as shown in Fig. 3d. Absorbing foams are applied to prevent the reflection from the environment. Due to the limited space of the microwave scanner, we have only measured the electric field distributions in the regions that are marked by the dotted boxes in Fig. 3a, b. The experimental results are plotted in Fig. 3e, f, respectively, for cases without and with the GAM. It is seen that they agree well with the simulation results shown in Fig. 3a, b. Therefore, the dielectric surface is indeed made invisible by the designed GAM. The discrepancy between the field distributions of the numerical and experimental results is mainly attributed to the following two reasons. The first one is the fabrication error (<0.1 mm) in the structure of the GAMs. The second one is a tiny air gap of around 0.2 mm between the dielectric and the upper aluminum plate of the parallel-plate waveguide, which is introduced to scan the field in the dielectric side in the experiment (see details in Supplementary Note 4 and Supplementary Fig. 5). Despite these experimental disturbances, the main directions of transmission in the numerical and experimental results are clearly consistent with each other as well as the direction of incidence.

The proposed GAM in Figs. 2 and 3 is designed for a specific incident angle (45°). However, when the incident angle is changed within the range from −60° to 60°, we find the functionality of antireflection is well maintained, although the angle of refraction is different from the incident angle (see details in Supplementary Note 5 and Supplementary Figs. 6-7).

### Invisible curved surface of a high-index dielectric

**Fig. 4:**
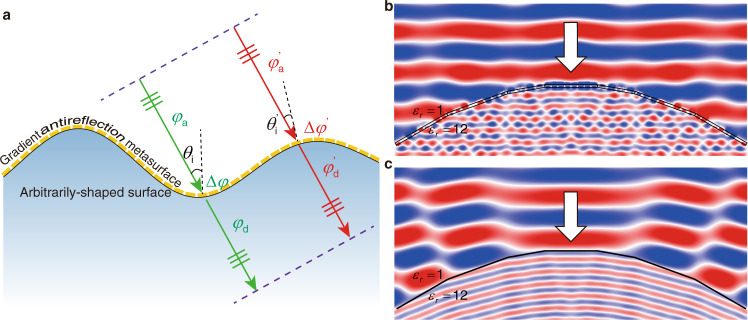
Invisible curved surface. **a** Schematic of the arbitrarily-shaped invisible surface enabled by GAMs. Two dashed lines represent the wavefront of the incident and transmitted waves separately. Green and red arrows depict two light rays impinging on the GAM at different locations. **b**, **c** Simulated E_z_ field distributions for a curved dielectric surface with (**b**) and without (**c**) the designed GAM under the illumination of a TE-polarized wave.

We emphasize that the principle of GAMs is general and applicable to any corrugated or curved surfaces. As schematically shown in Fig. 4a, a curved surface can also be made “invisible” by covering it with a GAM consisting of subwavelength antireflection meta-atoms. The reflection on the interface can be eliminated while the transmitted waves can be manipulated to propagate in the same direction of the incidence. Therefore, the phase accumulation between two reference planes of the incident and transmitted waves, which are depicted by the dashed lines in Fig. 4a, should be a constant for all propagating rays, i.e.,

2 $${\varphi }_{a}+\varDelta \varphi +{\varphi }_{d}={{Const}}.$$

where *φ*_*a*_ and *φ*_*d*_ depict the phase accumulation in the optical paths within the backgrounds of the incidence and transmission sides, respectively. In other words, the phase shift induced by the GAM should compensate for the difference in the optical paths of different rays.

In order to demonstrate this principle, we design a GAM that can make a curved surface (bump) of a high-index material with *ε* = 12 invisible. The curved surface is arbitrarily chosen and discretized by segments with an angular resolution of 10°. Therefore, the corresponding GAM structure is composed of anti-reflecting and phase-configurable meta-atoms designed for four different incident angles, i.e. 0°, 10°, 20°, and 30°. The electric field distribution obtained by FDTD simulations is plotted in Fig. 4b, where almost no reflection is observed and the wavefront of the transmitted waves indicates that the main direction of transmission is the same as that of incidence. This confirms the invisibility of the curved surface. For contrast, Fig. 4c shows the electric field distribution of a bare surface with obvious reflection and focusing effect in transmission. The detailed geometry and configuration of GAM are shown in Supplementary Notes 6–8 and Supplementary Figs. 8–13.

### Wide and interesting applications based on the principle of the GAMs

Besides the realization of the invisible surface demonstrated here, a series of interesting applications can be realized through the GAMs. In the following, we demonstrate several more examples.

**Fig. 5:**
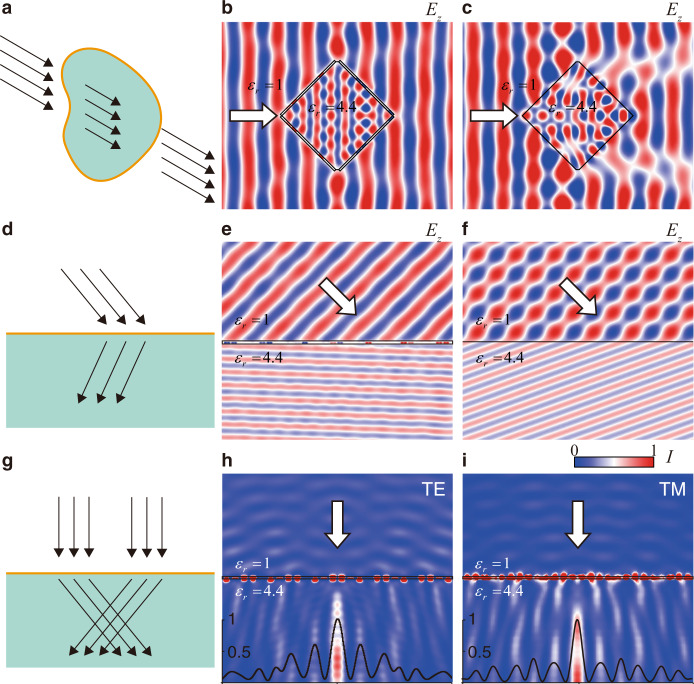
Several phenomena and reflection-less devices enabled by GAMs. **a** Schematic diagram of an invisible dielectric object by covering it with GAM. **b**, **c** Simulated electric field distributions of a diamond dielectric object covered with (**b**) and without (**c**) the GAM. **d** Schematic diagram of reflection-less negative refraction on a dielectric surface. **e**, **f** Simulated electric field distributions of a dielectric surface with (**e**) and without (**f**) the GAM. **g** Schematic diagram of a reflection-less flat axicon on a dielectric surface. **h**, **i** Simulated distributions of intensity for the designed reflection-less flat axicon under illumination of TE (**h**) and TM (**i**) polarized incident waves. The solid curves in (**h**) and (**i**) depict the normalized intensity along the bottom boundary of (**h**) and (**i**), respectively. White arrows depict the incident waves. Relative permittivity of the dielectrics is 4.4. GAMs in **b** and **e** are composed of the meta-atoms shown in Fig.2. Details of GAMs in **h** and **i** are shown in Supplementary Information.

Firstly, by covering the whole surface of a dielectric object with subtly designed GAMs, we can make the dielectric object invisible or “cloaked”, as shown in Fig. 5a. Here, a diamond-shaped dielectric object is utilized, as shown in Fig. 5b. Again, the same meta-atoms in Fig. 2 are applied, and the calculated electric field distribution confirms the status of invisibility. For contrast, Fig. 5c illustrates the scattering of the bare dielectric object, which exhibits strong scattering effect. Therefore, the GAMs could “cloak” dielectric objects.

Secondly, we demonstrate the phenomenon of reflection-less negative refraction on dielectric surfaces, as is schematically shown in Fig. 5d. This is achieved by using the same meta-atoms in Fig. 2 to construct a GAM with a phase gradience of −*ξ*, which is opposite to the case of Fig. 3. The angle of refraction is obtained as $\beta =\arcsin (({k}_{i}^{//}-\xi )/({n}_{g}{k}_{0}))=-1.89^\circ$. The electric field distributions in Fig. 5e, f confirm the reflection-less negative refraction, as well as the reflection and different refraction on a bare dielectric surface, respectively.

Thirdly, we demonstrate a reflection-less flat axicon for dielectrics by designing another set of meta-atoms and GAMs according to the same principle demonstrated above. Such an axicon can focus waves into a Bessel beam^9^ inside a dielectric medium with the perfect efficiency, as schematically shown in Fig. 5g. Since there is no reflection, all the incident energy can be transferred into the dielectric. Besides, the designed antireflection axicon works for both polarizations because all the meta-atoms have the *C*_4*v*_ symmetry. The focusing functionality and the polarization independence are numerically demonstrated in Fig. 5h, i, which show the simulated intensity distributions of the designed reflection-less flat axicon under normal incidence of TE and TM polarizations, respectively. Both of them match well with the expected function of axicon. The intensity distribution across the center of the generated Bessel beam is also plotted in the inset of Fig. 5h, i. More details of this reflection-less flat axicon are shown in Supplementary Notes 9–10 and Supplementary Figs. 14–16.

## Discussion

We note that the GAMs demonstrated here are tailored to the surface shape, the material parameters and the properties of the incident waves like the incident angle, frequency, and polarization. By employing the abundant degrees of freedom in the multi-layer design, we are able to realize the GAMs for both polarizations and enhance operating bandwidth by further structure optimization^11–13,45^. The other limitations could also be relieved by exploring active control in the metasurface design^14,24^, which has now been widely explored.

With the coalescence of antireflection and wavefront controllability in a thickness of the deep subwavelength scale, the concept of GAMs has gone beyond the traditional antireflection coatings and metasurfaces, and thus potentially, has significant impacts on many other subject areas. Besides the above examples, applications of GAMs may include the elimination of unwanted reflection in meta-lens systems, the exploration of high-index substrates for electromagnetic and optical transmission-type metasurfaces, and the enhancement of radiation efficiency for wave emitters, etc.

Precise manipulation of light or waves on the interfaces in the deep subwavelength scale is important. The ultra-small thicknesses of the GAMs ensure that the metasurfaces can have a close proximity to the interface, and thus can tailor the functionalities according to the subwavelength feature of the interface. On the contrary, traditional antireflection coatings with thicknesses comparable to or even larger than the wavelength are incapable of resolving such subwavelength details.

In conclusion, in this work we demonstrate a multi-layer metasurface which merges the two vital functions of anti-reflection and wavefront manipulation. The rich degrees of freedom endowed with the multi-layer design of the meta-atoms bestow the ability to break the phase-locking effect in single-layer antireflection coatings. While at the same time, the total thickness of the metasurfaces is still kept in a deep subwavelength scale, far below the quarter wavelength. Coalescence of antireflection and wavefront control is thus achieved in a single metasurface. One intriguing application of such metasurfaces is the realization of invisible surfaces in the transmission geometry, which is experimentally demonstrated here. Such an invisibility effect also works for surfaces of complicated geometries and high-index contrast, and can be extended to many other functionalities. Our discovery will open a new horizon for ultrathin interface devices that allow flexible and high-efficiency coupling between different media.

## Methods

### Numerical simulations

The full wave simulations are performed using CST Microwave Studio. In Fig.2, unit cell boundary is applied in *x* and *y* directions. Two Floquet ports are set in *z* direction to calculate S-parameters. In Figs. 3–5, periodic boundary condition is set in y direction and open boundary condition is set in *x* and *z* directions to reduce the reflection.

### Experimental apparatus

The samples were fabricated by the circuit board printing process. In the microwave experiments, the fabricated metasurface and dielectric blocks are assembled inside a parallel-plate waveguide composed of two flat aluminum plates. A standard X-band waveguide and a dielectric convex lens are used to generate a beam with finite width. The lower metal plate along with the samples was mounted by a stepper motor. The electric field was measured via an antenna fixed in a hole in the upper metal plate. The emitting X-band waveguide and the probing antenna are connected to a KEYSIGHT N5224B network analyzer to acquire the transmitted magnitude and phase of microwave signals.

## Supplementary information

Supplementary Information

## Data Availability

The data that support the findings of this study are available from the corresponding author upon reasonable request.
