# Supplementary material for: Invisible surfaces enabled by the coalescence of anti-reflection and wavefront controllability in ultrathin metasurfaces
Source: Nat Commun. 2021 Jul 26;12:4523. doi: 10.1038/s41467-021-24763-9 (PMC8313714; doi:10.1038/s41467-021-24763-9)
Supplement: Supplementary file 1 — Supplementary Information [file 41467_2021_24763_MOESM1_ESM.pdf]

Supplementary Information for
Invisible surfaces enabled by the coalescence of anti-reflection and wavefront controllability in ultrathin metasurfaces

Hongchen Chu[1,2], Haoyang Zhang[2], Yang Zhang[1], Ruwen Peng[1], Mu Wang[1], Yang Hao[2,*], Yun Lai[1,*]

[1] National Laboratory of Solid State Microstructures, School of Physics, Nanjing University, Nanjing 210093, China.

[2] School of Electronic Engineering and Computer Science, Queen Mary University of London, London E1 4NS, U.K..

* Corresponding authors

Supplementary Note 1

Detailed geometries of the five meta-atoms of the GAMs for "invisible" surface and the fabrication of the GAMs

We designed five meta-atoms by adopting 3-layer structures as the building block of the gradient antireflection metasurface (GAM), where 3 layers are carefully designed 0.018 mm-thick metallic micro-structures separated by two 1mm-thick dielectric spacers (ε=4.4) as shown in Fig. 2c. Supplementary Figure 1 shows the detailed geometrical parameters of the metallic micro-structures. Fourfold symmetric split-ring resonators (SRRs) are adopted. The width of metal lines is $w = 1mm$. The out dimension, gap, and periodicity of the fourfold symmetric SRRs are separately *r*, $t = 0.8mm$, and $p = 7.73mm$.

The metasurfaces are fabricated by laminating two 1-mm-thick FR4 printed circuit boards. The two boards are separated by 0.018 *mm*-thick copper patterns of Layer 2 and are covered by Layer 1 and Layer 3. The relative permittivity of the dielectric boards is ε=4.4.

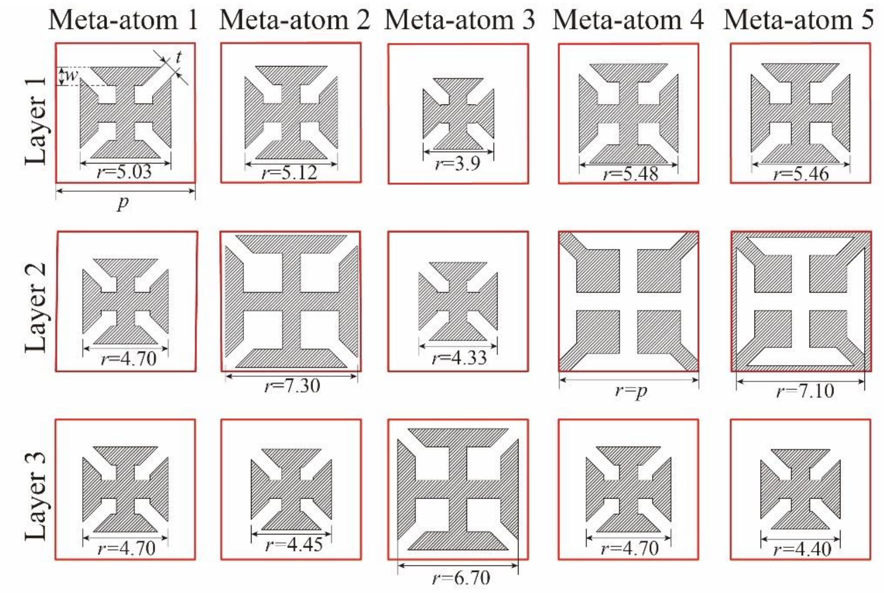

Supplementary Figure 1. Detailed geometries of the five meta-atoms of the GAM for "invisible" surface. The shadow areas depict copper. The unit of these numbers listed in the insets is mm.

Supplementary Note 2

GAMs composed of meta-atoms without metal sheets for meta-atom isolation

To illustrate the crucial effect of the meta-atom-isolation stemming from the metal sheets at the left and right sides of meta-atoms shown in Fig. 2c, we also design five meta-atoms without

metal sheets but possessing transmission properties similar to that with metal sheets. As shown in Supplementary Figures 2a and 2b, transmittances of the five meta-atoms are almost unity at central frequency i.e., 10 GHz, and the transmission phases at 10GHz are equally spaced and cover the full range of 2π. Though the designed five meta-atoms without metal sheets show similar properties as the ones with metal sheets, metasurface constructed of them fails in antireflection as can be found in the simulated electric field distribution shown in Supplementary Figure 2c, where the fluctuation of the wavefront in the incident side indicates the interference of the incidence and the considerable reflection. The underlying physics is that the mutual coupling between the neighboring meta-atoms causes them to deviate from the estimated transmission properties, which are calculated by setting periodic boundary conditions.

Detailed geometries of the five meta-atoms without metal sheets are shown in Supplementary Figure 3. The out dimension and periodicity of the fourfold symmetric SRRs are separately *r* and *p*=7.73 mm. Line widths of meta-atom 1 and meta-atom 2-5 are set as w_1=1.2mm and w_2=0.8mm separately. Two 1.5-mm-thick dielectric boards with relative permittivity ε=4.4 are separated by copper patterns of Layer 2 and are covered by Layer 1 and Layer 3. The thickness of the copper patterns is $0.018mm$.

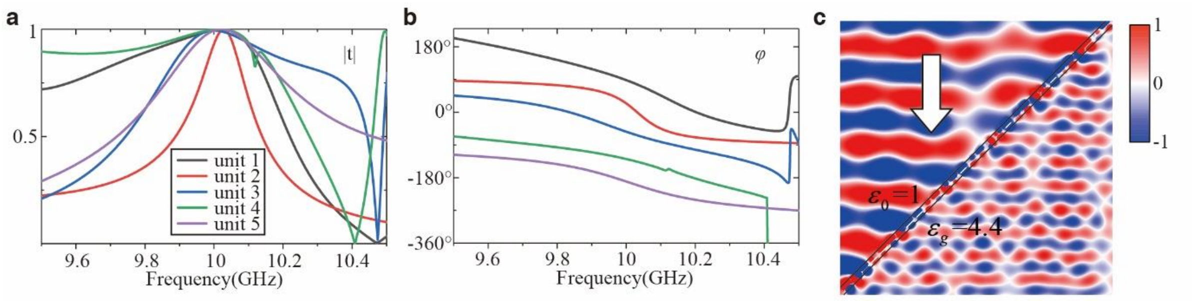

Supplementary Figure 2. a, **b** Simulated spectra of transmittance (a) and transmission phase (b) for the five meta-atoms without metal sheets. **c** Simulated E_z field distributions for the designed GAM without metal sheets, which is located on the surface of a dielectric material with $\varepsilon_g = 4.4$. The white arrow depicts a transverse-electric polarized incident wave impinging on the GAM with the incident angle of $45°$.

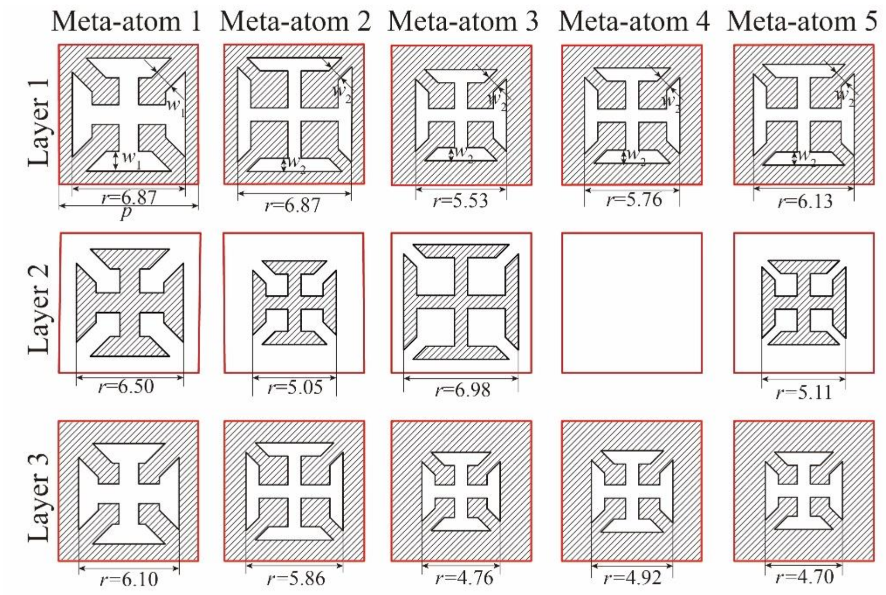

Supplementary Figure 3. Detailed geometries of the five meta-atoms without metal sheets. The shadow areas depict copper. The unit of these numbers listed in the insets is mm.

Supplementary Note 3

Bandwidth of the GAM in Fig.2

From Fig. 2, we find that in the frequency range from 9.92GHz to 10.07GHz, the transmittances of all the five meta-atoms are greater than 90% and the transmission phases are kept almost equidistant. The field distributions of the GAM at 9.92GHz and 10.07GHz are simulated and shown in Supplementary Figure 4, both of which indicate good antireflection function and the designed effect of "invisible surface", which are in sharp contrast with the results obtained without GAMs. Therefore, the working bandwidth of the GAMs is at least 0.15GHz. The narrow bandwidth of the GAM stems from the intrinsic resonance nature of the meta-atoms. In the future, some approaches such as combining PB phase and resonances, superimposing multiple resonant modes and dispersion compensation may be utilized to widen the bandwidth of the GAMs.

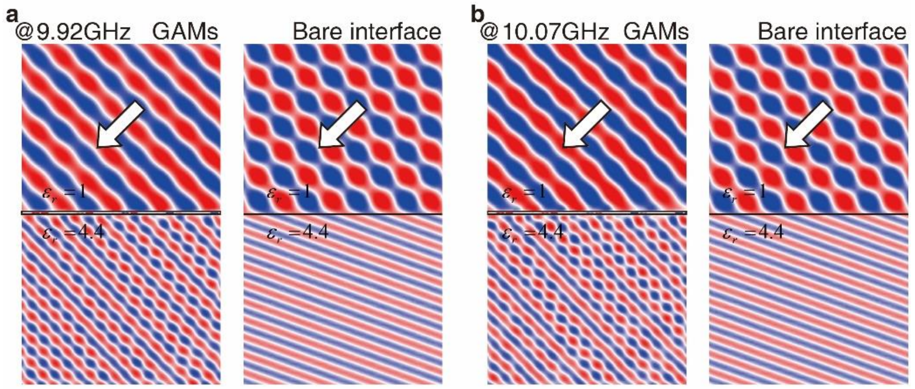

Supplementary Figure 4. Simulated E_z field distributions for a dielectric surface with (left panel) and without (right panel) the designed GAM under the illumination of a TE polarized wave at frequencies of 9.92GHz (a) and 10.07GHz (b).

Supplementary Note 4

Several factors rendering imperfection in the simulation results and discrepancy in the experimental results.

The small fluctuation in the wavefront of transmitted waves in Fig. 3(b) is mainly attributed to the discretization of the metasurface as well as the coupling between neighboring units, as has been previously observed in other transmission-type metasurfaces[27,29]. Better results may be achieved by increasing the number of meta-atoms in a supercell.

We have analyzed the underlying causes of the discrepancy between the field distributions of the numerical and experiment results, and we find two main reasons. The first one is the inevitable fabrication error in the structure of the GAMs. The metal patterns in the GAMs are fabricated by a printed-circuit-board (PCB) etching machine with a tolerance of $\pm 0.1mm$. Through numerical simulations, we find such fabrication errors may slightly affect the performance of the designed GAMs. In the simulation results shown in Supplementary Figure 5a, the field disturbance is increased by the random errors between $-0.1mm$ and $0.1mm$ imposed on the side length (r) of the split-ring resonators (SRRs) in the GAMs, as shown in Supplementary Figure 1.

The second reason is the tiny air gap of around $0.2mm$ between the samples and the upper aluminum plate of the parallel-plate waveguide in the experiments. In practice, it is not possible to scan the field inside the dielectric slab, therefore, a small air gap is sandwiched between the dielectric slab and the upper aluminum plate, and the field is scanned within the gap via a moving detector. However, this air gap has some influences on the results. By combining the air gap and the random fabrication errors together in the simulation model, we obtained the electric field distribution as shown in Supplementary Figure 5b. A clear disturbance in the field distribution is observed. Especially, the disturbance in both the transmitted and reflected wavefronts is increased substantially, which is quite similar to the measured results shown in the main text. Nevertheless, although there is some disturbance in the field distribution, the

direction of the transmission is evidently consistent with the direction of incidence, which proves the "invisible surface" phenomenon.

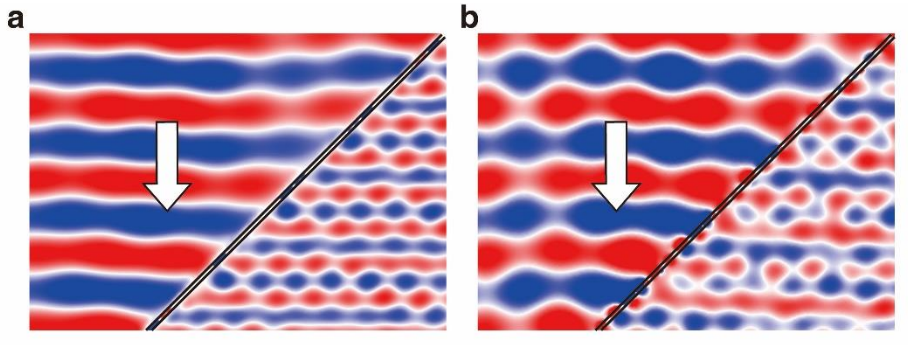

Supplementary Figure 5. Simulated E_z field distributions for a dielectric surface with the designed GAM under the illumination of a TE polarized wave with the incident angle of $45°$. (a) Random errors between -0.1*mm* to 0.1*mm* are imposed on the side length of the split-ring-resonators in the designed GAM. (b) An air gap of 0.2*mm* between the samples and the upper aluminum plate of the parallel-plate waveguide in experiments is taken into consideration.

Supplementary Note 5

Incident-angle tolerance of the proposed GAMs.

The proposed GAM meta-atoms in Fig. 2 were designed for a specific incident angle ($45°$). However, when the incident angle is changed within the range from $-60°$ to $60°$, we find that the functionalities of the designed meta-atoms, i.e. the antireflection effect and designed transmission phase, are both well maintained. In Supplementary Figure 6, we plot the calculated transmittance and transmission phase of the five meta-atoms in Fig. 2 as the functions of the incident angles. For almost all incident angles within $60°$, the high transmittance and transmission phases of the meta-atoms are almost unchanged. In Supplementary Figure 7, we plot the simulated field distribution for the GAM under the incident angles of $-60°$, $-30°$, $0°$, $30°$ and $60°$. Surely, the "invisible surface" effect disappears at other incident angles due to the difference between the angles of incidence and transmission. But the antireflection and wave refraction effects are clearly observed. However, in Supplementary Figure 6 we also see that for large incident angles, e.g., $75°$, the transmittance of the five meta-atoms is reduced significantly. Hence the anti-reflection functionality of the GAM fails for such large incident angles.

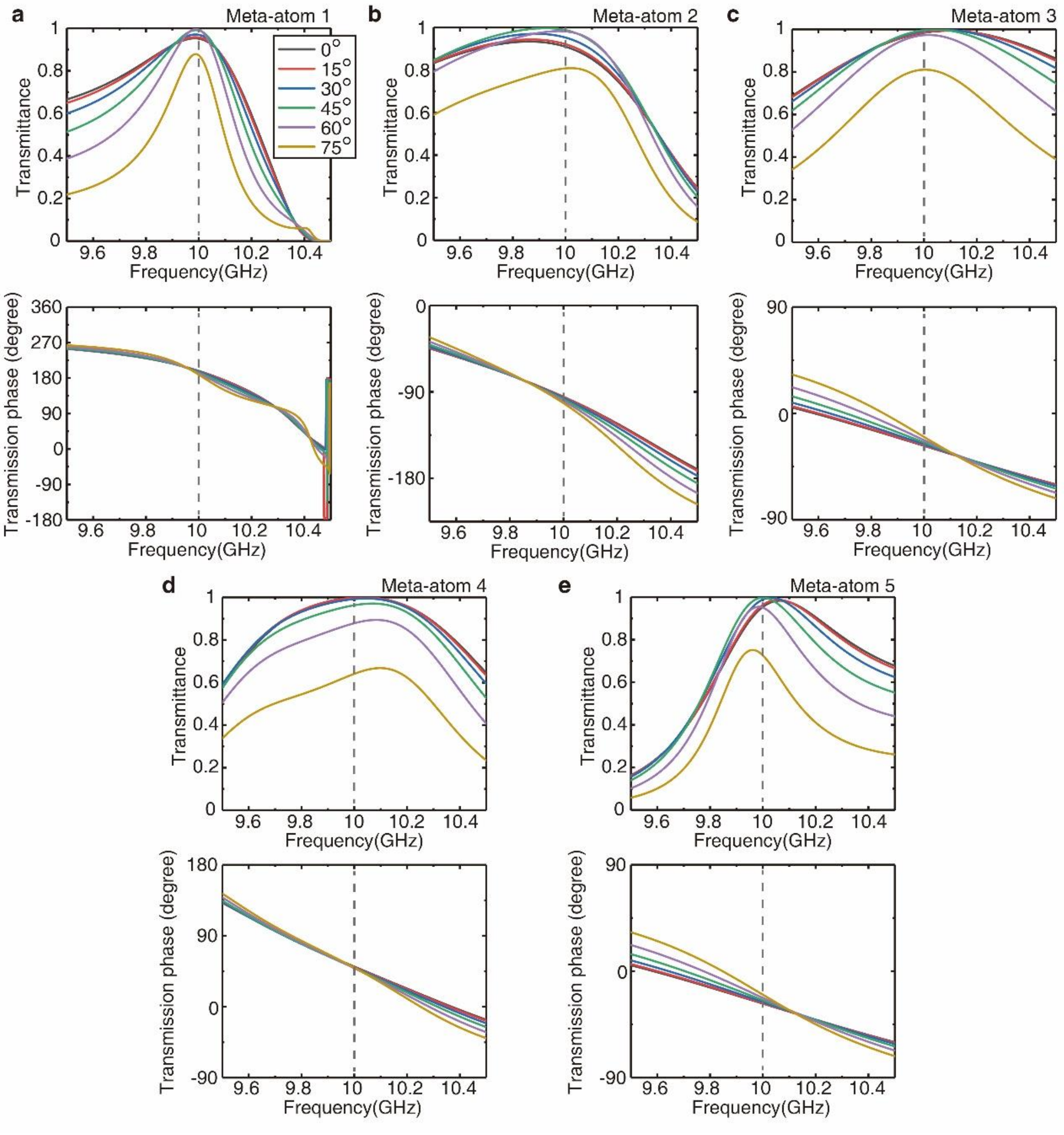

Supplementary Figure 6. Simulated results of transmittance and transmission phase spectra of the meta-atoms of GAMs in Fig. 2 for incidence at various incident angles.

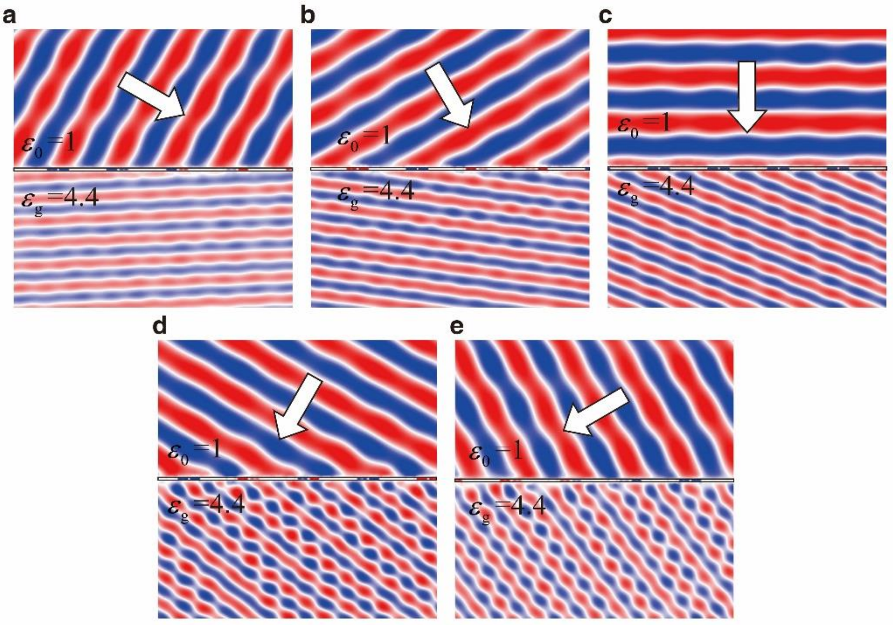

Supplementary Figure 7. Simulated results of field distributions of the meta-atoms of GAMs designed at $45°$ under illumination of TE polarized incidence at incident angles of $-60°$, $-30°$, $0°$, $30°$ and $60°$.

Supplementary Note 6

Invisible interface with high permittivity contrast enabled by GAMs.

In order to demonstrate the generality of our approach, we have designed the GAMs for the materials with a much higher relative permittivity $\varepsilon = 12$. We choose this permittivity because it is comparable to that of silicon in the infrared regime, as well as that of some common low-loss ceramics like alumina (8-11.5) in the microwave regime. Without the antireflection coatings, such a high-index material would lead to a large reflection (say, 36% for TE polarized incidence with an incident angle of $30°$). Moreover, the wavelength is reduced by 3.46 times in the material with $\varepsilon = 12$, comparing to that in free space. The total reflection induced by the high index has been widely applied in the field of silicon photonics to form waveguides and other devices. Therefore, the application of GAMs to the materials with $\varepsilon \leq 12$ should cover most of the practical applications.

In Supplementary Figure 8, we demonstrate the realization of "invisible surface" for a dielectric medium with relative permittivity $\varepsilon = 12$. The effect of the GAM is clear and evident. In Supplementary Figure 8a, the designed GAM makes the TE-polarized incident wave with an incident angle of 30° transmit straightly through the dielectric surface of $\varepsilon = 12$ without any reflection or refraction, just as if the surface turns invisible. While in Supplementary Figure 8b, without the GAM, the phenomena of reflection and refraction are both clearly seen. Detailed geometries of the GAM are shown in Supplementary Figure 9.

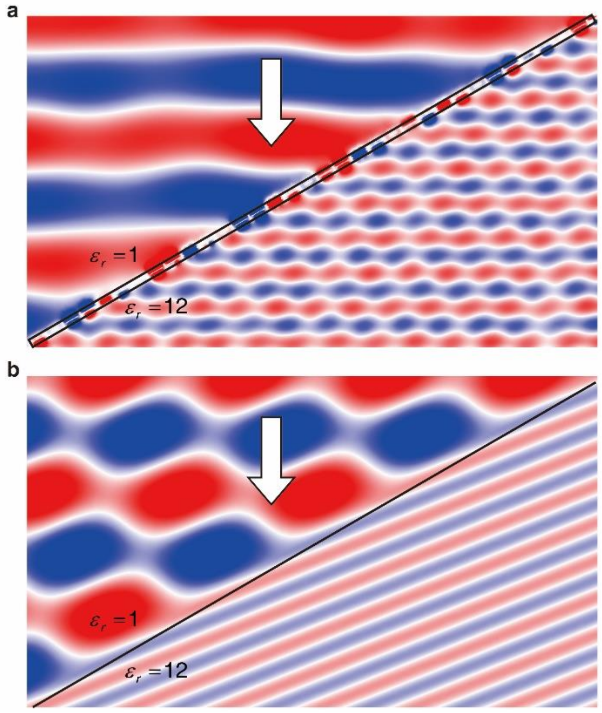

Supplementary Figure 8. Simultaneous elimination of the reflection and refraction effects on an air-dielectric interface with the permittivity contrast of 1:12 by the GAM. **a,b** Simulated E_z field distributions for the dielectric surface with (a) and without (b) the designed GAM under the illumination of a TE polarized wave with the incident angle of 30°.

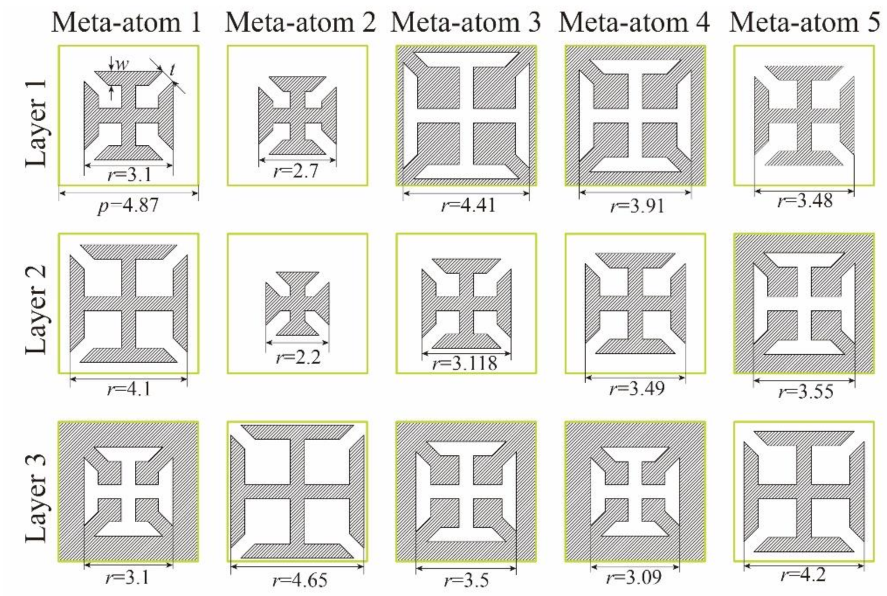

Supplementary Figure 9. Detailed geometries of the five meta-atoms working for 30-degree incidence in the invisible surface in Fig. 4. The side lengths of the SRRs along y-axis are set as $r_y = r_x$. Both w and t are set as 0.5. The shadow areas depict copper. The unit of these numbers listed in the insets is mm.

Supplementary Note 7

Detailed geometries of GAMs of the curved invisible surface in Fig. 4.

The detailed geometries of GAMs of invisible surface in Fig. 4 working for incidence with incident angles of 0°, 10°, 20° and 30° are shown in Supplementary Figures 10-12 and Supplementary Figure 9 respectively. The side lengths along the y-direction of all the meta-atoms designed for 0°, 10°, 20° and 30° incidences are set as 4.87 mm while the side lengths along the x-direction of meta-atoms designed for 0°, 10°, 20° and 30° are separately set as 4.87 mm, 7 mm, 5.93 mm, and 4.87 mm. The thickness and relative permittivity of the dielectric spacer between the three metallic layers are separately 1 mm and 12.

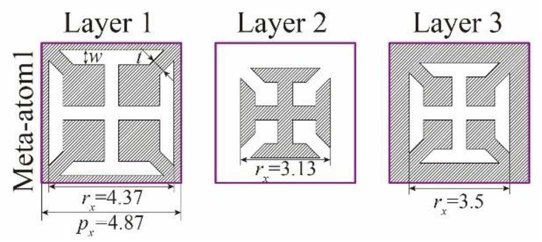

Supplementary Figure 10. Detailed geometries of the meta-atom working for normal incidence in the invisible surface in Fig. 4. The side lengths of the SRRs along y-axis are set as $r_y = r_x$. Both w and t are set as 0.5. The shadow areas depict copper. The unit of these numbers listed in the insets is mm.

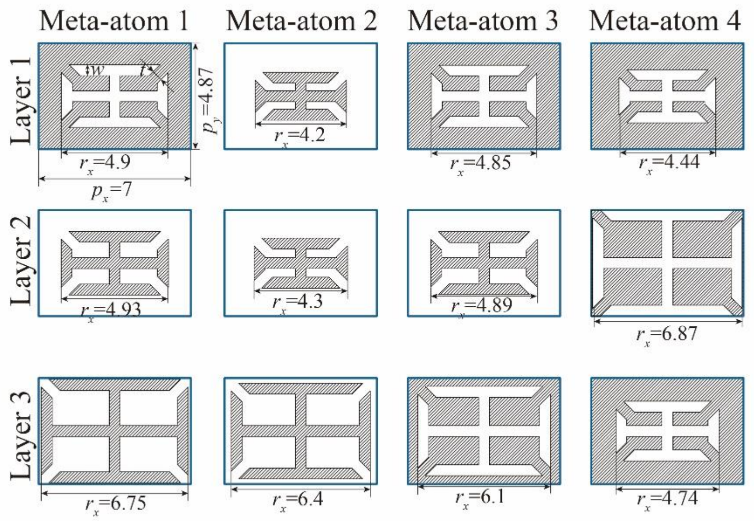

Supplementary Figure 11. Detailed geometries of the five meta-atoms working for 10 degrees incidence in the invisible surface in Fig. 4. The side lengths of the SRRs along y-axis are set as $r_y = r_x - 2$. Both w and t are set as 0.5. The shadow areas depict copper. The unit of these

numbers listed in the insets is mm.

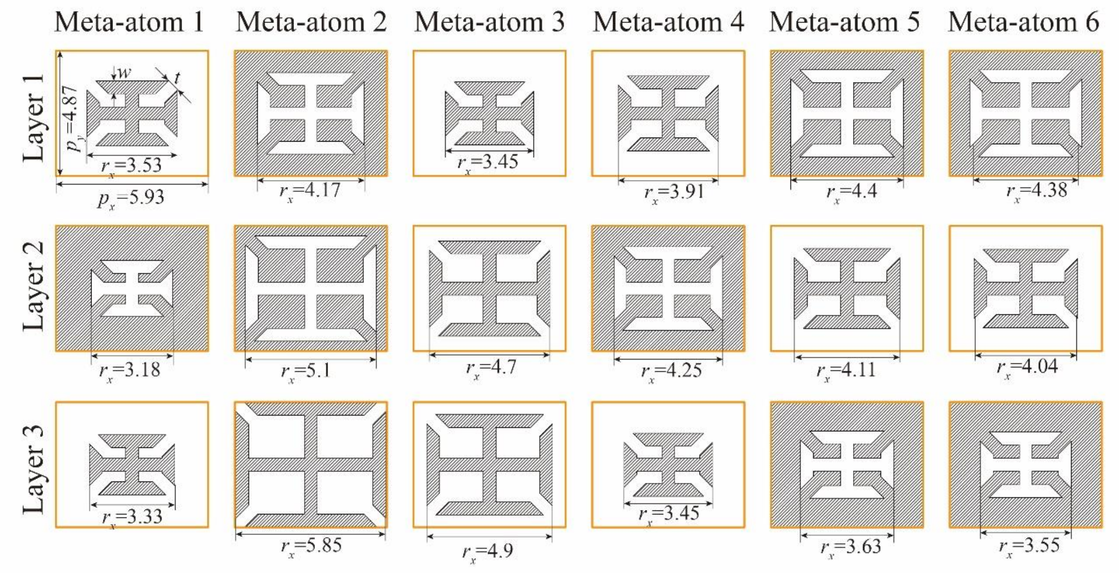

Supplementary Figure 12. Detailed geometries of the five meta-atoms working for 20 degrees incidence in the invisible surface in Fig. 4. The side lengths of the SRRs along y-axis are set as $r_y = r_x - 1$. Both *w* and *t* are set as 0.5. The shadow areas depict copper. The unit of these numbers listed in the insets is mm.

Supplementary Note 8

Detailed configurations of GAMs in applications in the main text.

The detailed configurations of GAM in applications in Fig. 4b, Fig.5b, Fig.5e, and Fig.5h are separately shown in Supplementary Figures 13a-13d. The purple, blue, yellow, and green frames in Supplementary Figure 13a represent metasurface designed for 1-12 interface and incident angles of 0°, 10°, 20°, and 30° respectively. The red frames in Supplementary Figures 13b and 13c and black frames Supplementary Figure 13d represent metasurface designed for 1-4.4 interface and incident angles of 45° and 0°. The numbers depict the corresponding meta-atoms.

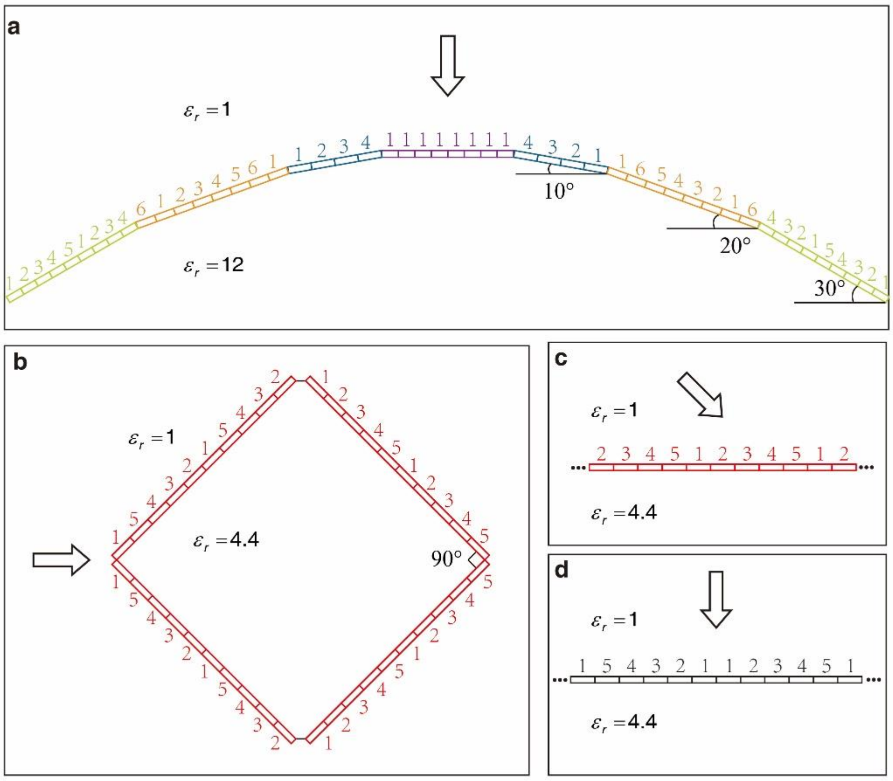

Supplementary Figure 13. Detailed configurations of the GAM-induced invisible surface (a), invisibility cloak (b), negative refraction (c), and antireflection axicon (d) reported in the main text.

Supplementary Note 9

Meta-atoms of reflection-less flat axicon based on GAMs.

To construct the GAMs that function as a reflection-less flat axicon, we have designed five meta-atoms, as shown in Supplementary Figure 14a. They are composed of three layers of metallic patterns separated by two thin dielectric spacers with the relative permittivity $\varepsilon_r = 4.4$ and a thickness of $1mm$. The length and total thickness of the meta-atoms are $p = 7.7mm$ and $d = 2mm$, respectively. By Finite-Difference Time-Domain (FDTD) simulations, we have calculated the transmittance and transmission phase spectra of these meta-atoms, which is shown in Supplementary Figure 14b. It is observed that at the working frequency $f = 10GHz$, these meta-atoms exhibit both high transmittance and gradually changing transmission phase. The detailed geometric parameters of these five meta-atoms are shown in Supplementary Figure 15, where the shadow areas depict copper which is a perfect electric conductor in the microwave regime. The out dimension and line width of the fourfold symmetric split-ring resonators are separately r and $w = 0.8mm$. The gap of the split-ring resonators is set as $t = 0.8mm$.

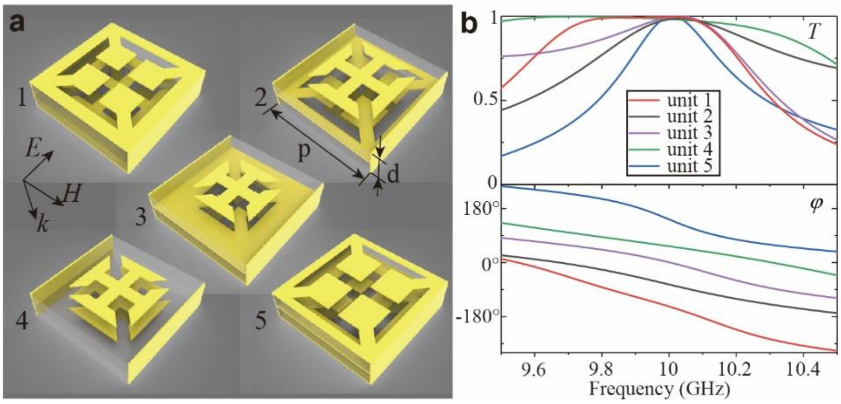

Supplementary Figure 14. Meta-atoms of the reflection-less flat axicon. a Schematic of the five designed meta-atoms, which are all composed of 3-layer metal structures separated by two 1-mm-thick dielectric spacers. The side length and thickness are separately $p = 7.7mm$ and $d = 2mm$. Metal sheets are used on the left and right sides of each meta-atom to reduce the mutual coupling. **b** Simulated spectra of transmittance (upper panel) and transmission phase (lower panel) of the five meta-atoms.

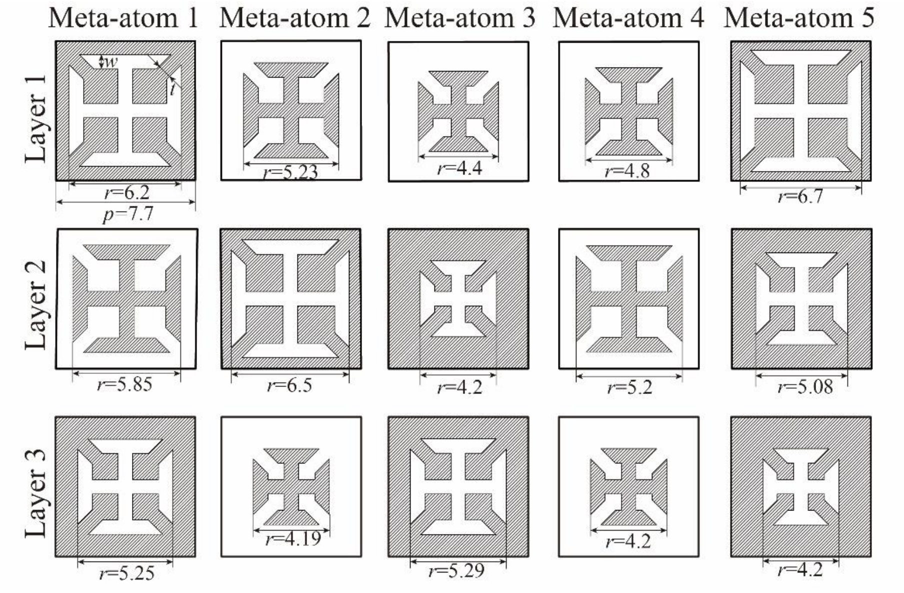

Supplementary Figure 15. Detailed geometries of the five meta-atoms in the reflection-less flat axicon. The shadow areas depict copper. The unit of these numbers listed in the insets is mm.

Supplementary Note 10

Field distributions of the antireflection axicon under TE and TM polarized incidence.

Supplementary Figure 16 shows the simulated electric field distributions of the designed

antireflection axicon, which is shown in Fig. 5, under TE-polarized incidence (a) and the simulated magnetic field distributions of the axicon under TM-polarized incidence (b). We note that the transmission efficiencies of the antireflection axicon for TE and TM polarized incidence are very close, though the magnetic field in Supplementary Figure 16b seems to be enhanced more greatly than the electric field in Supplementary Figure 16a. The reason for the greater enhancement of the magnetic field is that the energy flux of the electromagnetic waves is proportional to $\sqrt{\mu / \varepsilon} H^2$. Even for unity transmission, the magnetic field in the dielectric object is enhanced to meet the conservation of energy.

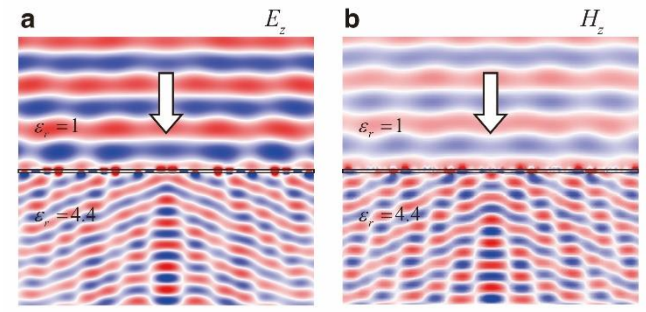

Supplementary Figure 16. a Electric field distribution of the designed antireflection axicon shown in Fig. 5 under TE-polarized incidence. **b** Magnetic field distribution of the axicon under TM-polarized incidence.
